# Longitudinal assessment of quality assurance measurements in a 1.5 T MR‐linac: Part II—Magnetic resonance imaging

**DOI:** 10.1002/acm2.13586

**Published:** 2022-03-25

**Authors:** Ergys Subashi, Alex Dresner, Neelam Tyagi

**Affiliations:** ^1^ Department of Medical Physics Memorial Sloan Kettering Cancer Center New York New York USA; ^2^ Philips Healthcare MR Oncology Cleveland Ohio USA

**Keywords:** longitudinal, MR‐linac, 1.5T, quality assurance

## Abstract

**Purpose:**

To describe and report longitudinal quality assurance (QA) measurements for the magnetic resonance imaging (MRI) component of the Elekta Unity MR‐linac during the first year of clinical use in our institution.

**Materials and methods:**

The performance of the MRI component of Unity was evaluated with daily, weekly, monthly, and annual QA testing. The measurements monitor image uniformity, signal‐to‐noise ratio (SNR), resolution/detectability, slice position/thickness, linearity, central frequency, and geometric accuracy. In anticipation of routine use of quantitative imaging (qMRI), we characterize B0/B1 uniformity and the bias/reproducibility of longitudinal/transverse relaxation times (T1/T2) and apparent diffusion coefficient (ADC). Tolerance levels for QA measurements of qMRI biomarkers are derived from weekly monitoring of T1, T2, and ADC.

**Results:**

The 1‐year assessment of QA measurements shows that daily variations in each MR quality metric are well below the threshold for failure. Routine testing procedures can reproducibly identify machine issues. The longitudinal three‐dimensional (3D) geometric analysis reveals that the maximum distortion in a diameter of spherical volume (DSV) of 20, 30, 40, and 50 cm is 0.4, 0.6, 1.0, and 3.1 mm, respectively. The main source of distortion is gradient nonlinearity. Maximum peak‐to‐peak B0 inhomogeneity is 3.05 ppm, with gantry induced B0 inhomogeneities an order of magnitude smaller. The average deviation from the nominal B1 is within 2%, with minimal dependence on gantry angle. Mean ADC, T1, and T2 values are measured with high reproducibility. The median coefficient of variation for ADC, T1, and T2 is 1.3%, 1.1%, and 0.5%, respectively. The median bias for ADC, T1, and T2 is −0.8%, −0.1%, and 3.9%, respectively.

**Conclusion:**

The MRI component of Unity operates within the guidelines and recommendations for scanner performance and stability. Our findings support the recently published guidance in establishing clinically acceptable tolerance levels for image quality. Highly reproducible qMRI measurements are feasible in Unity.

## 1 INTRODUCTION

The integrated MR‐linac^1^, ^2^, ^3^, ^4^ provides a novel platform for the delivery of precision radiotherapy by allowing for the acquisition of high spatiotemporal resolution magnetic resonance imaging (MRI) images with increased sensitivity and specificity to soft tissue anatomy. The increased accuracy in target and organ‐at‐risk delineation has enabled treatment methods with ablative doses and improved outcomes.^5^, ^6^, ^7^, ^8^, ^9^ Treatment precision, accuracy, and efficiency will be further enhanced by automatic segmentation,^10^ staging,^11^ and outcome modeling.^12^

The Elekta Unity MR‐linac (Elekta AB, Stockholm, Sweden) couples a diagnostic 1.5 T MRI scanner (Philips Healthcare, Best, Netherlands) with a 7‐MV linear accelerator. While commissioning and quality assurance (QA) procedures for each separate system are described in several national and international reports,^13^, ^14^, ^15^, ^16^, ^17^ currently there are no published consensus protocols specific to the hybrid machine. The MR‐linac presents with challenges that are not encountered when each component is considered separately. The clinical implementation of this device requires a review and revision of acceptance, commissioning, and QA methods to address differences within its subcomponents.^18^, ^19^, ^20^, ^21^ A recent publication by a consortium of clinical users, developers, and manufacturers provides recommendations for QA procedures in the Elekta Unity system.^22^

The high‐field MR‐linac further enables the implementation of imaging biomarkers for radiotherapy treatment planning and response assessment. The validation of prognostic and predictive biomarkers depends primarily on a detailed characterization of measurement bias and uncertainty.^23^, ^24^, ^25^, ^26^ The feasibility of quantitative MRI (qMRI) in Unity has been demonstrated by early adopters of the system reporting on the accuracy and repeatability of longitudinal/transverse relaxation rate and apparent diffusion coefficient (ADC).^27^, ^28^, ^29^ Recent work has also described the feasibility of metabolic imaging with chemical exchange saturation transfer MRI.^30^

The longitudinal assessment of QA measurements provides necessary information about machine performance, stability, and safety. The analysis of these data offers further guidance in establishing clinically acceptable tolerance levels for anatomic, functional, and metabolic imaging. In this work, we report the 1‐year assessment of relevant imaging QA measurements for the MRI component of the Elekta Unity system. The analysis of the QA measurements for the linac component of Unity has recently been published.^21^

In anticipation of routine use of quantitative imaging, we also provide baseline measurements for several global machine parameters and describe the bias and uncertainty for three of the most commonly used qMRI biomarkers (T1/T2/ADC).

## 2 MATERIALS AND METHODS

### 2.1 Daily assessment of image quality

**FIGURE 1:**
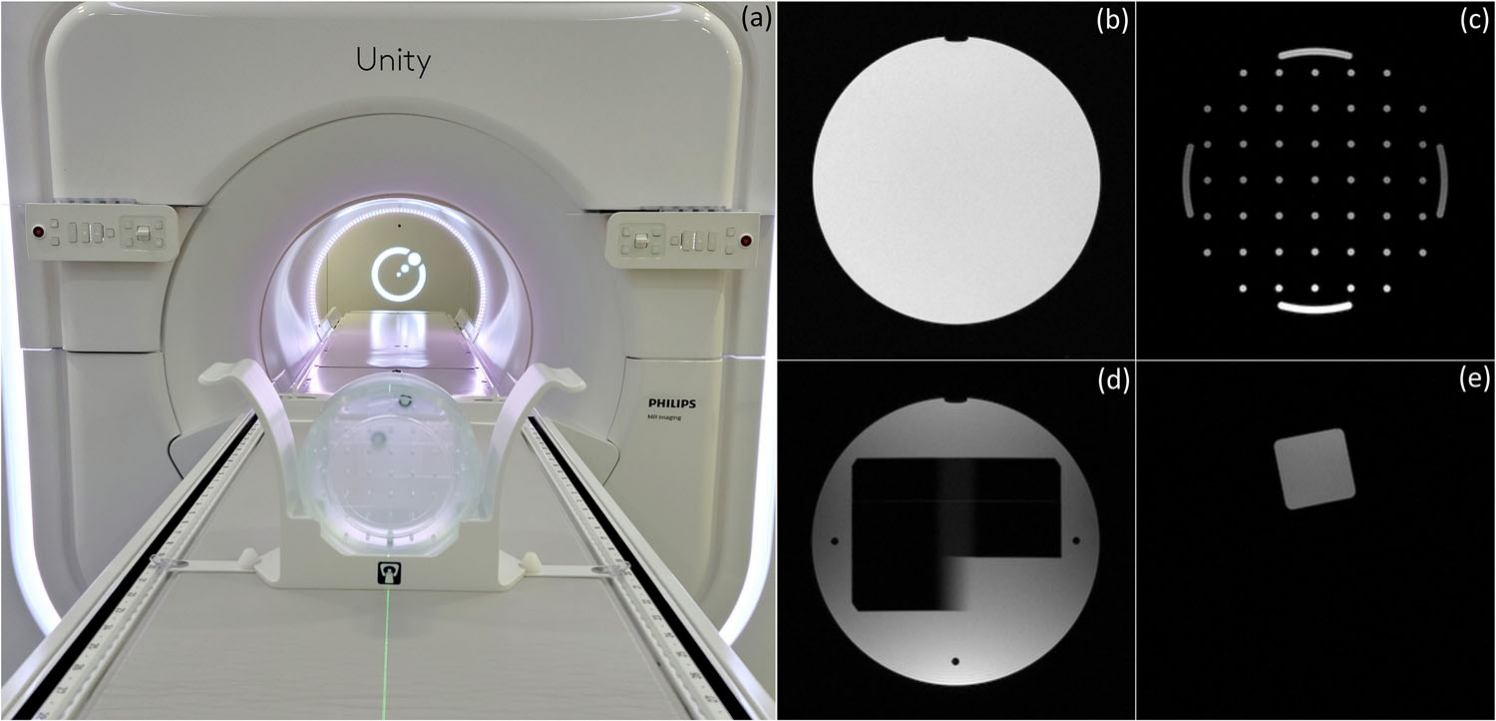
Periodic Image Quality Test (PIQT) is used to monitor daily image quality. (a) PIQT phantom inside positioning cradle. For clarity, anterior coil is not shown. Representative images used in the calculation of (b) signal‐to‐noise ratio (SNR) and uniformity, (c) spatial linearity, (d) slice profile, and (e) spatial resolution

The quality of MRI images is monitored daily using the measurements reported in the Periodic Image Quality Test (PIQT), as provided by the vendor. PIQT uses a phantom with a known geometry to characterize the signal‐to‐noise ratio (SNR), uniformity, resolution, slice profile, linearity, and central frequency. The phantom and representative images used in calculating these QA metrics are shown in Figure 1. The analysis is based on the MRI standards published by the National Electrical Manufacturers Association (NEMA).^31^, ^32^, ^33^ Image quality is assessed for three of the most commonly used pulse sequences: multi‐slice (MS) multi‐echo spin‐echo (MESE), MS fast field echo (FFE), and two‐dimensional (2D)‐MESE. The quadrature body coil is evaluated separately from the combined body/surface coil used in transmit/receive mode. The surface coil consists of a four‐element anterior array mounted on an adjustable bridge and a four‐element posterior array mounted in the bore electronics underneath the treatment couch. Table 1 lists the main acquisition parameters for the sequences used to measure image quality with PIQT. All sequences use Cartesian sampling.

**TABLE 1:** Acquisition parameters for the pulse sequences used to measure image quality with Periodic Image Quality Test (PIQT)

Imaging sequence	Tx coil	Rx coil	TR/TE (ms)	Acq voxel (mm^3^)
MS‐MESE (two echoes)	Body	Ant/Post	1000/30/100	1.20 × 1.20 × 5.00
MS‐FFE (single echo)	Body	Ant/Post	200/15	1.20 × 1.20 × 5.00
2D‐MESE (three echoes)	Body	Body	1000/50/100/150	0.98 × 0.98 × 15.00

Abbreviations: 2D‐MESE, two‐dimensional multi‐echo spin‐echo; Acq, acquisition; MS‐FFE, multi‐slice fast field‐echo; MS‐MESE, multi‐slice multi‐echo spin‐echo; Tx/Rx, transmit/receive.

#### 2.1.1 Signal‐to‐noise and uniformity

The SNR and uniformity are measured for all three sequences listed in Table 1. SNR is estimated as the ratio of the mean signal in a central region of the phantom divided by the noise in the background.^31^ Uniformity is calculated by comparing the maximum and minimum signal intensity in a large region of the phantom^32^:

(1) $$\mathrm{Uniformity}=\frac{\max-\min}{\max+\min}\times 100\%$$

Figure 1b shows a representative image used in the calculation of SNR and uniformity. The tolerance levels used in the tests that monitor these QA metrics are listed in Table 2.

**TABLE 2:** Tolerance levels used in the Periodic Image Quality Test (PIQT) tests that monitor signal‐to‐noise ratio (SNR) and uniformity

Imaging sequence	SNR tolerance	Uniformity tolerance (%)
MS‐MESE	Echo 1: SNR > 59	Echo 1: uniformity < 47
	Echo 2: SNR > 44	Echo 2: uniformity < 48
MS‐FFE	SNR > 48	Uniformity < 47
2D‐MESE	Echo 1: SNR > 45	Echo 1: uniformity < 10
	Echo 2: SNR > 39	Echo 2: uniformity < 10
	Echo 3: SNR > 30	Echo 3: uniformity < 10

Abbreviations: 2D‐MESE, two‐dimensional multi‐echo spin‐echo; MS‐FFE, multi‐slice fast field‐echo; MS‐MESE, multi‐slice multi‐echo spin‐echo.

#### 2.1.2 Spatial linearity

Spatial linearity is determined by comparing the known and measured phantom dimensions along eight radial directions. Figure 1c shows a representative image used to calculate linearity. This section of the phantom contains 45 holes of 5 mm diameter arranged in a grid with 25 mm spacing. The size of the grid is 150 × 150 mm^2^. Linearity is used as an aggregate measure of image distortion and is calculated for each radial direction by:

(2) $$\mathrm{Linearity}=\frac{d_{\mathrm{Measured}}-d_{\mathrm{Actual}}}{d_{\mathrm{Actual}}}\times 100\%$$

where $d_{i}$ denotes the dimension of the phantom. Note that this metric characterizes only in‐plane geometric distortion and provides limited information on its spatial distribution. Linearity is evaluated with the MS spin‐echo sequence using a tolerance of 0.5%.

#### 2.1.3 Slice profile

The slice profile is determined by analyzing an image across two opposing wedges of known dimensions (known slopes). The line profile across the wedge‐pair projected onto the imaging plane is used to calculate the full width at half maximum (FWHM) and slice integral.^33^ Figure 1d shows a representative image used to estimate these metrics. The slice profile provides an aggregate measure of the RF‐excitation chain (body coil) and gradient performance. It is monitored only for the MS spin‐ and gradient‐echo sequences with the tolerance levels listed in Table 3.

**TABLE 3:** Tolerance levels used in the Periodic Image Quality Test (PIQT) tests that monitor slice profile

Imaging sequence	FWHM (mm)	Slice integral (mm)
MS‐MESE	Echo 1: 4.65–5.15	Echo 1: 4.85–5.35
	Echo 2: 4.45–4.95	Echo 2: 4.65–5.15
MS‐FFE	4.75–5.25	4.90–5.40

Abbreviations: FWHM, full width at half maximum; MS‐FFE, multi‐slice fast field‐echo; MS‐MESE, multi‐slice multi‐echo spin‐echo.

#### 2.1.4 Spatial resolution

Spatial resolution is quantified using the pixel size calculated from the line spread function along the edges of the phantom shown in Figure 1e. Pixel size is measured in the horizontal (frequency encoding) and vertical (phase encoding) direction for both echoes of the MS‐MESE imaging sequence. Resolution is an aggregate measure of system performance and is affected by multiple components in the imaging chain, including hardware (receive/transmit coils, gradients) and software (reconstruction) implementation. The frequency and phase encoding direction have different tolerance levels, as shown in Table 4.

**TABLE 4:** Tolerance levels used in the Periodic Image Quality Test (PIQT) test that monitors spatial resolution

Imaging sequence	Horizontal pixel (mm)	Vertical pixel (mm)
MS‐MESE	Echo 1: <1.3	Echo 1: <1.5
	Echo 2: <1.3	Echo 2: <1.5

*Note*: Frequency encoding is in the horizontal direction, phase encoding is in the vertical direction.

Abbreviation: MS‐MESE, multi‐slice multi‐echo spin‐echo.

#### 2.1.5 Central frequency

The central (resonant) frequency is measured with all three imaging sequences in PIQT. For consistency, we present the central frequency reported from the MS‐MESE sequence. The measured frequency is compared to the nominal frequency calculated using the nominal field of 1.5 T and the proton gyromagnetic ratio *γ* = 42.577478518 MHz/T.^34^ This test is primarily used for preventative inspection as the drift in central frequency is expected to be minimal, particularly with the use of the zero boil‐off cryostat that preserves the helium level in Unity.^22^

### 2.2 Distortion

**FIGURE 2:**
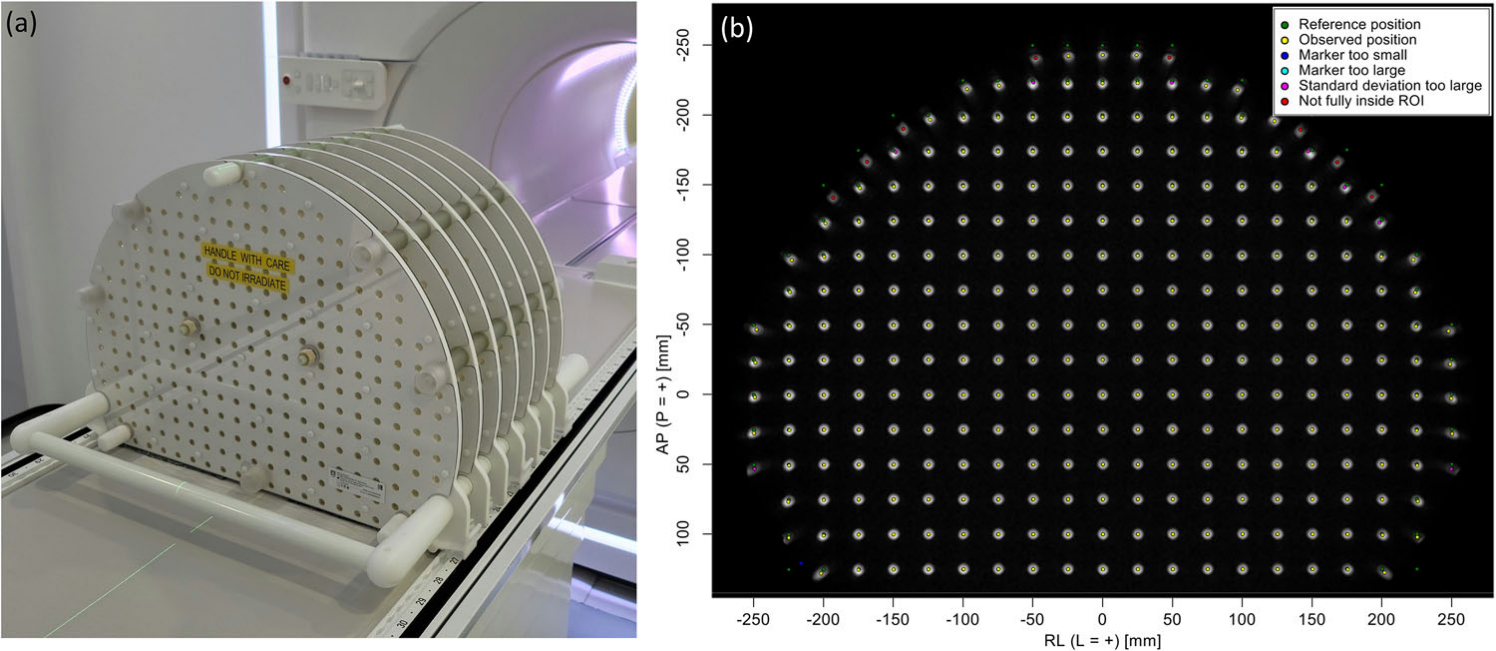
The spatial distribution of geometric distortion is measured with a three‐dimensional (3D) phantom of fiducials at known positions. (a) Phantom positioning on treatment couch. (b) Representative image from one of the plates with color‐coded markers derived from the analysis

The spatial distribution of geometric distortion is measured using a 3D phantom consisting of seven flat plates with 1932 markers at precisely machined positions. The markers are placed in a grid spaced by 25 mm in‐plane and 55 mm across slices. The central plate of the 3D phantom is positioned at the isocenter of the scanner. Figure 2 shows the phantom on the treatment couch and a representative image used in estimating geometric distortion. The phantom, imaging sequence (T1‐weighted 3D‐FFE), and the software that performs the analysis are provided by the vendor. Geometric distortion is calculated at the location of each marker as the difference between the measured and known coordinates of the fiducials. The maximum value of distortion in a spherical volume of diameter (diameter of spherical volume, DSV) 20, 30, 40, and 50 cm is monitored longitudinally. The spatial distribution of distortion is also generated as a heatmap and can be used for further analysis. The QA test was performed weekly during the first 3 months of clinical use, monthly thereafter, and if needed after machine service or upgrades. The tolerance levels are 1.0, 2.0, 4.0, and 20.0 mm for a DSV of 20, 30, 40, and 50 cm, respectively.

### 2.3 B0 and B1 homogeneity

**FIGURE 3:**
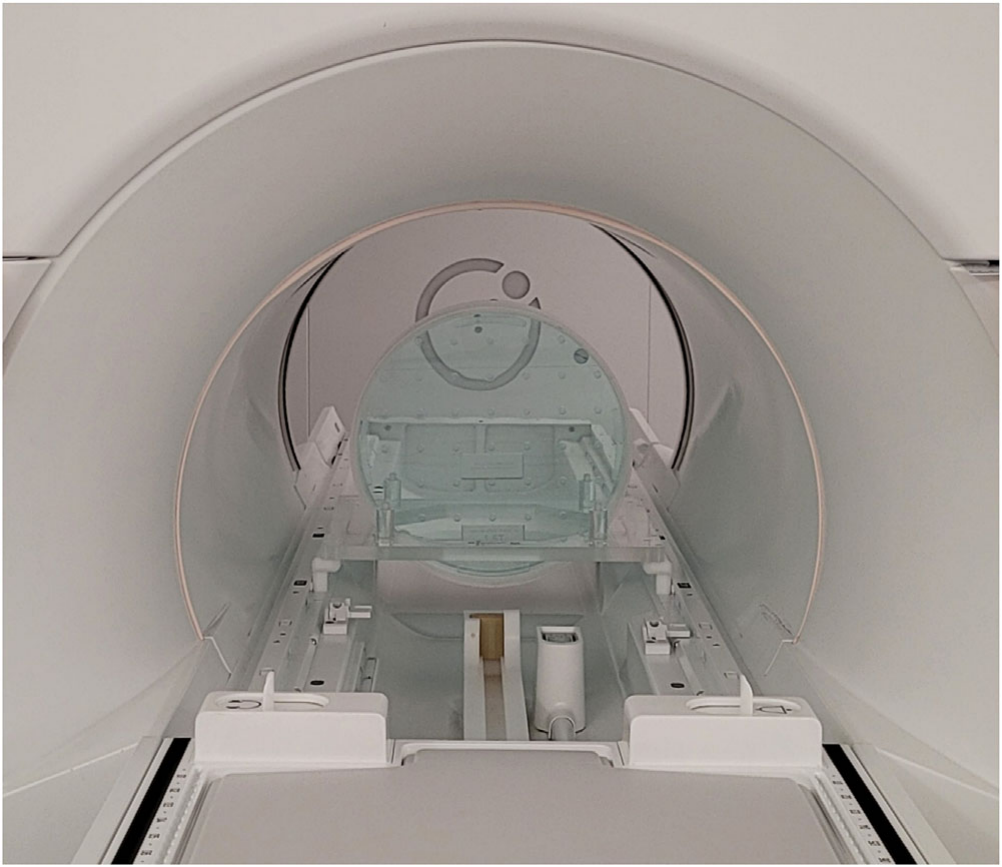
B0 and B1 homogeneity are characterized using a uniform cylindrical phantom, provided by the vendor. The phantom has a diameter of 40 cm and can be positioned at the isocenter using a custom‐made cradle

Static (B0) and excitation (B1) field homogeneity are critical requirements in every aspect of MRI and spectroscopy. In anticipation of routine use of quantitative imaging, we characterize B0 and B1 homogeneity using a uniform cylindrical phantom provided by the vendor. The phantom has a diameter of 40 cm and can be positioned at the isocenter using a custom‐made cradle, as shown in Figure 3. Measurements are performed in the transverse plane using the quadrature body coil in transmit and receive mode. To avoid edge effects, the analysis of B0 and B1 uniformity is performed over a 35‐cm diameter region‐of‐interest (ROI) placed at the phantom center. Field homogeneity is assessed at gantry angles 0°, 30°, 60°, 90°, 120°, 150°, 180°, 210°, 240°, 270°, 300°, 330°. Pre‐scan calibration measurements were only performed at gantry 0°. This simulates the worst‐case scenario when imaging has begun at one gantry angle and continues as the gantry is rotated for treatment delivery. The gantry dependence of B0 uniformity is reported using the peak‐to‐peak difference, defined as the range of the values in the B0‐map. The mean B0‐map across all gantry angles is subtracted from each measurement to remove the static B0 contribution. The gantry dependence of B1 uniformity is reported using the average deviation from the nominal B1. Note that while this phantom is not designed for estimating the B1 distribution, the setup provides a convenient way for simultaneously characterizing B0 and B1.

B0‐maps are acquired using a dual‐echo 2D‐FFE sequence with the following imaging parameters: TR/TE1/TE2 = 30/5.4/6.9 ms, flip angle (FA) = 20°, number of averages = 4, acquisition matrix = 150 × 150, field of view = 450 × 450 mm^2^, slice thickness = 10 mm, slice spacing = 15 mm, number of slices = 3 (axial), total acquisition time = 1 min, 12 s. The maps are reconstructed in the scanner and exported offline for further analysis. B0 values are expressed in Hz as a deviation from the central frequency and converted to parts per million (ppm) using ppm = ΔB0/B0 × 10^6^.

B1‐maps are acquired using a dual‐TR 3D‐FFE sequence with the following imaging parameters: TR1/TR2/TE = 30/150/2.0 ms, FA = 60°, number of averages = 2, acquisition matrix = 100 × 100, field of view = 400 × 400 mm^2^, slice thickness = 12 mm, slice spacing = 6 mm, number of slices = 6 (axial), total acquisition time = 1 min, 40 s. The maps are reconstructed in the scanner and exported offline for further analysis. B1 values are expressed as a fraction of the nominal RF power needed to achieve the prescribed FA.

### 2.4 Quantitative MRI

We study the bias and reproducibility of the three most commonly used quantitative MRI biomarkers: longitudinal relaxation rate (T1), transverse relaxation rate (T2), and ADC. The bias (B) is defined as:

(3) $$B=\frac{D_{\mathrm{Measured}}-D_{\mathrm{Nominal}}}{D_{\mathrm{Nominal}}}\times 100\%$$

where D represents T1, T2, or ADC.

Reproducibility is quantified using the percent coefficient of variation (COV):

(4) $$\mathrm{COV}=\frac{\sigma }{\mu }\times 100\%$$

where σ and μ represent the standard deviation and the mean value of the measured data.

#### 2.4.1 Quantitative relaxometry

**FIGURE 4:**
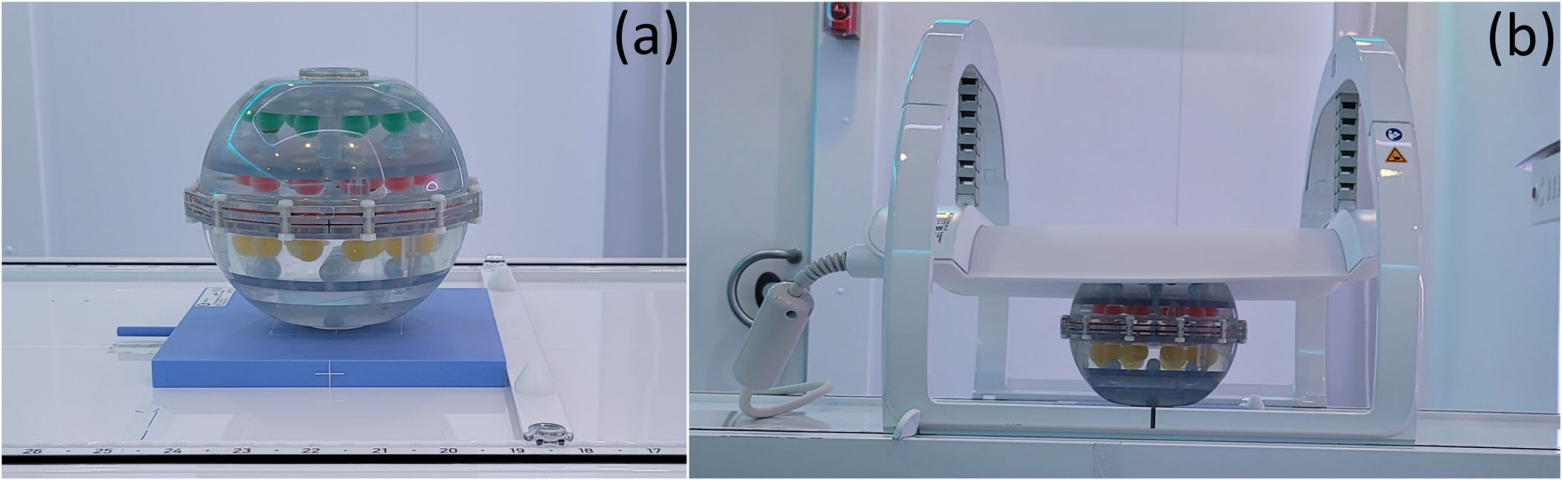
The National Institute of Standards and Technology (NIST)/International Society of Magnetic Resonance in Medicine (ISMRM) phantom used to assess the bias and reproducibility of the longitudinal and transverse relaxation rate. (a) Phantom positioned on the treatment couch. (b) The anterior coil was placed flush with the surface of the phantom to ensure reproducibility and maximize signal‐to‐noise ratio (SNR)

The bias and reproducibility of relaxometry measurements is assessed using the phantom developed by the National Institute of Standards and Technology (NIST) and International Society of Magnetic Resonance in Medicine (ISMRM).^35^ Figure 4 shows the NIST/ISMRM phantom positioned on the treatment couch with the anterior coil placed flush on its surface. The phantom includes two parallel plates containing 14 spherical vials with known relaxation rates, listed in Table A1. The nominal relaxation rates were compared to the measured values with weekly scans over the course of 4 weeks.

##### Longitudinal relaxation rate (T1)

The magnitude of the spoiled steady‐state FFE sequence is approximated by:

(5) $$S\;\left(\alpha ,\mathrm{TR},\mathrm{TE}\right)=S_{0}\;\frac{\sin\left(\alpha \right)\left(1-e^{-\mathrm{TR}/T1}\right)}{1-\cos\left(\alpha \right)e^{-\mathrm{TR}/T1}}$$

where *α* is the FA and *S*_0_ is proportional to the equilibrium longitudinal magnetization and the system gain function. Equation (5) can be linearized voxel‐wise as:

(6) $$\begin{array}{ccc} \frac{S(\alpha ,\mathrm{TR})}{\sin(\alpha )} & = & E1\frac{S(\alpha ,\mathrm{TR})}{\tan(\alpha )}+S_{0}\left(1-E1\right)\\ E1 & = & \exp\left(-\frac{\mathrm{TR}}{T1}\right) \end{array}$$

A varying flip angle (VFA) acquisition with *α_i_* = {*α*_1_, …, *α_N_*} and fixed TR is used to measure *S*(*α_i_*). The T1 and *S*_0_ maps are obtained by solving:

(7) $$\begin{array}{ccc} \vec{y} & = & m\cdot \vec{x}+b\\ y_{i} & = & \frac{S_{i}}{\sin(\alpha_{i})},\;x_{i}=\frac{S_{i}}{\tan(\alpha_{i})},\;i=1,\text{…},N\\ m & = & \;E1,b=S_{0}\;\left(1-E1\right) \end{array}$$

The following imaging parameters are used in the measurement of T1: TR/TE = 20/2.3 ms, FA = {4°, 22°}, number of averages = 1, acquisition matrix = 160 × 160, field of view = 320 × 320 mm^2^, slice thickness = 3 mm, slice spacing = 1.5 mm, number of slices = 67. The total acquisition time per FA is ∼45 s. The set of FAs used in the VFA sequence was estimated using the formalism by Deoni et al.^36^ The optimal FAs were calculated for T1 in the range 1000–2000 ms and the mean value for the lower and upper bounds was used in the acquisition. Equation (7) was solved on a voxel‐by‐voxel basis and the analysis was performed in a circular ROI of 1‐cm diameter centered on each vial of interest.

##### Transverse relaxation rate (T2)

The magnitude of the MESE sequence is approximated by:

(8) $$S\left(t\right)=S_{0}\cdot \exp\left(-\frac{\mathrm{TE}}{T2}\right)$$

where *S*_0_ is proportional to the equilibrium longitudinal magnetization and the system gain function. Given a range of TE values, Equation (8) can be linearized voxel‐wise to obtain a solution for T2. The following imaging parameters are used in the measurement of T2: TR/TE/ΔTE = 4000/22/11 ms, eight echoes, number of averages = 1, acquisition matrix = 160 × 160, field of view = 320 × 320 mm^2^, slice thickness = 3 mm, slice spacing = 3 mm, number of slices = 33. To minimize the effect of stimulated echoes, a dummy echo is generated and discarded before the echo‐train used in the analysis. The total acquisition time is ∼5.5 min. Equation (8) is solved on a voxel‐by‐voxel basis and the analysis is performed in a circular ROI of 1‐cm diameter centered on each vial of interest.

#### 2.4.2 Diffusion‐weighted imaging

**FIGURE 5:**
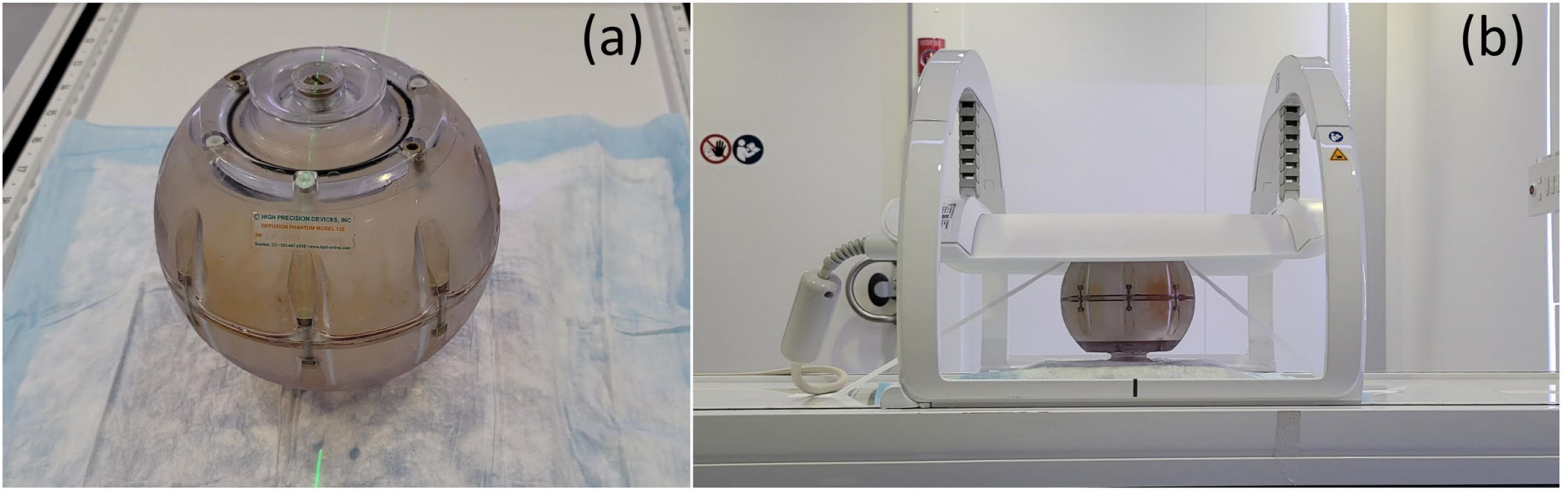
The Radiological Society of North America (RSNA)/National Institute of Standards and Technology (NIST)/International Society of Magnetic Resonance in Medicine (ISMRM) phantom used to assess the bias and reproducibility of the apparent diffusion coefficient. (a) Phantom positioned on the treatment couch. (b) The anterior coil was placed flush with the surface of the phantom to ensure reproducibility and maximize signal‐to‐noise ratio (SNR)

**FIGURE A1:**
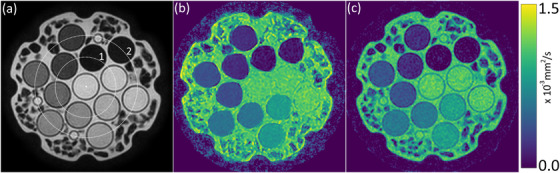
(a) T2‐weighted image of the diffusion phantom showing the inner and outer ring of vials labeled as (1) and (2), respectively. Each ring contains six vials with the concentration of polyvinylpyrrolidone (PVP) varying from 0% to 50%. White dot represents location of isocenter. The distance between the isocenter and the inner ring is approximately 35 mm. The distance between the isocenter and the outer ring is approximately 60 mm. (b) Apparent diffusion coefficient (ADC) map acquired with single‐shot EPI and imaging parameters listed in manuscript. (c) ADC map acquired with Cartesian spin‐echo and imaging parameters listed in manuscript

The quantitative diffusion phantom (CaliberMRI Inc., Boulder, CO, USA) developed in collaboration with the Radiological Society of North America (RSNA), NIST, and ISMRM, has been described in detail elsewhere.^37^ The phantom contains 13 cylindrical vials with varying concentrations of polyvinylpyrrolidone (PVP) in aqueous solution. The concentration of PVP and the respective nominal ADC values are listed in Table A2. The vials are arranged in two concentric rings such that each ring contains the entire range of PVP concentrations, as can be seen in Figure A1. Figure 5 shows the diffusion phantom positioned on the treatment couch with the anterior coil flush on its surface. The phantom is placed on top of a 4‐cm plastic plate to raise the vials to the isocenter. The vials are immersed in an ice‐water bath for at least 6 h prior to scanning and remain at 0°C for the duration of the experiments.

The signal intensity of the diffusion‐weighted acquisition is approximated by:

(9) $$S\left(b\right)=S_{0}\cdot \;\exp\left(-b\cdot \mathrm{ADC}\right)$$

where *S*(*b*) and *S*_0_ represent the signal intensity with and without diffusion weighting, respectively. The *b* value represents the diffusion‐weighting factor. Given a range of *b* values, Equation (9) can be linearized voxel‐wise to obtain a solution for ADC. Diffusion measurements were performed using a single‐shot spin‐echo EPI (SS‐EPI) imaging sequence with the following parameters: *b* = {0, 500, 900, 2000 s/mm^2^}, TR/TE = 15 000/149 ms, NEX = 1, acquisition matrix = 128 × 128 mm^2^, field of view = 200 × 200 mm^2^, slice thickness = 4 mm, number of slices = 25 (coronal plane), total acquisition time = 3 min. Equation (9) was solved on a voxel‐by‐voxel basis and the analysis was performed in a circular ROI of 1‐cm diameter centered on each vial of interest.

To demonstrate the effect of susceptibility artifacts that may arise with the SS‐EPI sequence, the experiment was repeated by switching to a Cartesian spin‐echo acquisition with the same imaging parameters as above except: TE = 277 ms (minimum), number of averages = 5.

### 2.5 ACR

Image quality is also evaluated using the phantom and measurement methods recommended by the American College of Radiology (ACR).^14^ The large ACR phantom is used to determine geometric accuracy, slice position accuracy, slice thickness, percent integral uniformity (PIU), percent signal ghosting (PSG), high‐contrast spatial resolution, and low‐contrast detectability. Acquisition parameters for the recommended T1/T2‐weighted sequences, definitions of QA metrics, and tolerance levels are described in the physics section of the ACR MRI QA manual.^14^ The analysis is performed at the console.

## 3 RESULTS

### 3.1 Daily assessment of image quality

The longitudinal trend of PIQT measurements is reported over the course of 1 year. The analysis includes the first measurement of each day unless a QA failure was found to be due to setup error rather than machine performance.

#### 3.1.1 Signal‐to‐noise and uniformity

**FIGURE 6:**
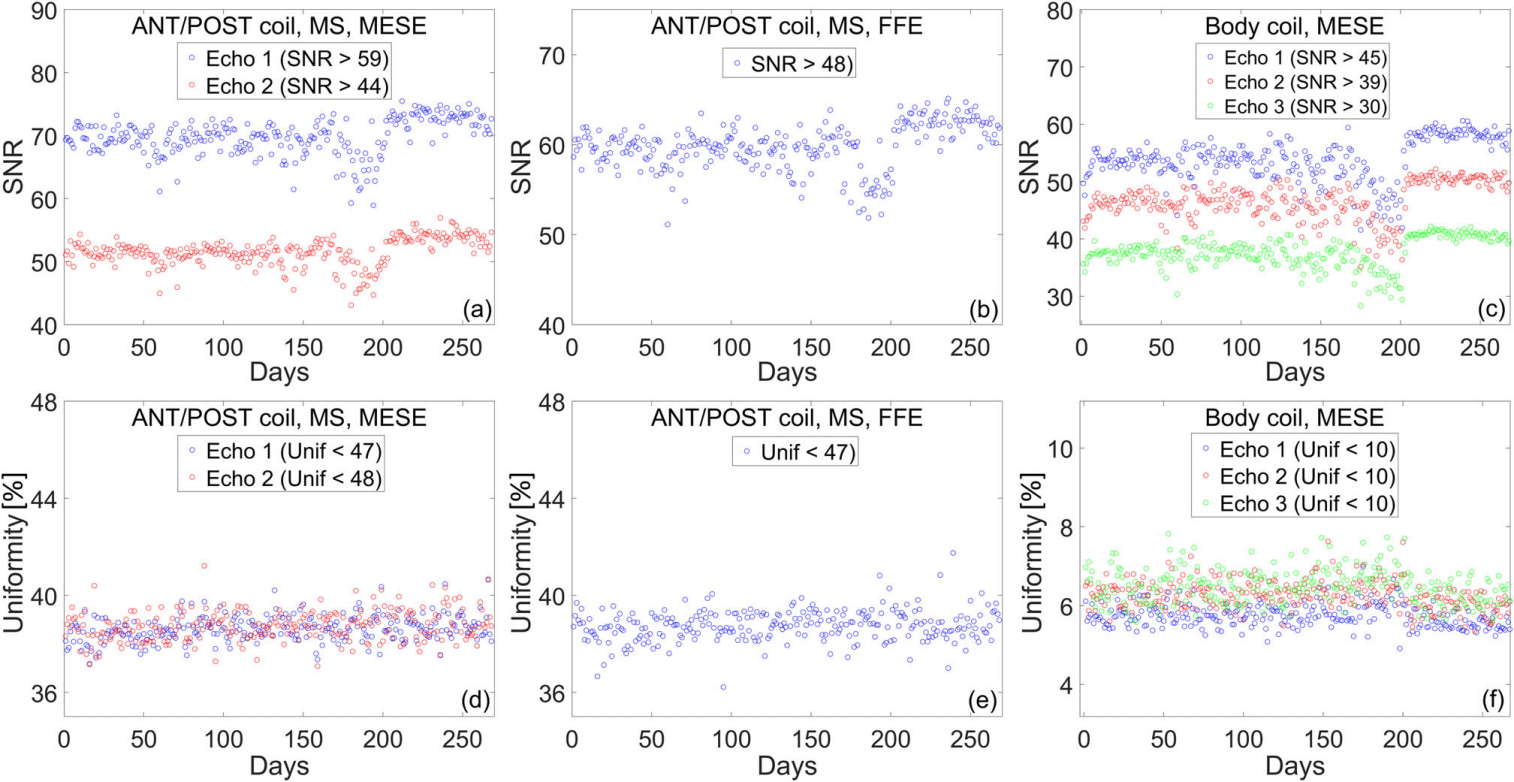
Longitudinal assessment of signal‐to‐noise ratio (SNR) and uniformity over the course of 1 year. Top row: SNR measurements for (a) multi‐slice multi‐echo spin‐echo (MS‐MESE) sequence using the body and surface coil, (b) multi‐slice fast field‐echo (MS‐FFE) sequence using the body and surface coil, and (c) MESE using the body coil. Bottom row: uniformity measurements for (d) MS‐MESE sequence using the body and surface coil, (e) MS‐FFE sequence using the body and surface coil, and (f) MESE using the body coil

**FIGURE A2:**
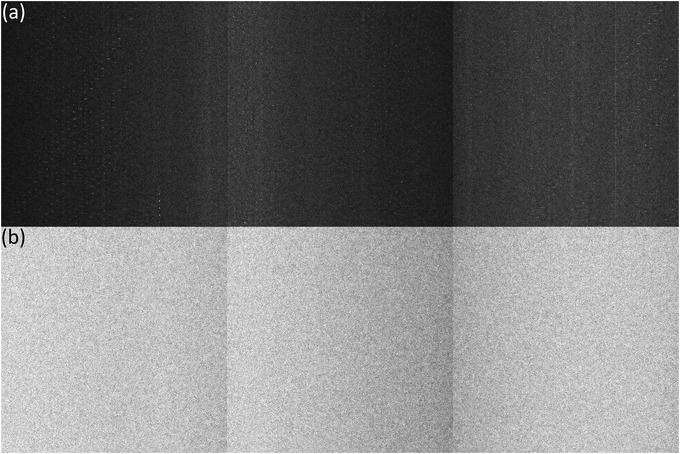
We observed a constant decrease in signal‐to‐noise ratio (SNR) starting at approximately day 160. (a) A noise scan revealed a consistent source of noise that appears as bright dots in a background of Rayleigh distributed noise. (b) Noise scans after the replacement of couch electronics show the expected Rayleigh distributed noise

Figure 6 shows the longitudinal trend of SNR and uniformity. The mean and standard deviation of the SNR and uniformity for the first 150 days of clinical use are given in Table 5. Based on our data, daily variations in SNR are on the order of ∼5%, as measured by the COV. This worst‐case value is for the third echo of the 2D‐MESE sequence. Daily variations in uniformity are on the order of ∼6%, as measured by the COV. Again, this worst‐case value is for the third echo of the 2D‐MESE sequence. Variations are much smaller for the other sequences. We observed a constant decrease in SNR starting at approximately day 160. A noise scan revealed a consistent source of noise which was identified to originate in the electric components of the treatment couch. After replacement of couch electronics, the SNR recovered to baseline values. Figure A2 shows the noise scans before and after machine service. Uniformity was not affected by the noise in the electric components of the couch.

**TABLE 5:** Mean and standard deviation of the signal‐to‐noise ratio (SNR) and uniformity over the course of the first 150 days

Imaging sequence	SNR (*μ* ± *σ*)	Uniformity (*μ* ± *σ*) (%)
MS‐MESE	Echo 1: 69.3 ± 2.0	Echo 1: 38.6 ± 0.5
	Echo 2: 51.1 ± 1.5	Echo 2: 38.6 ± 0.6
MS‐FFE	59.3 ± 1.8	38.7 ± 0.6
2D‐MESE	Echo 1: 53.3 ± 2.2	Echo 1: 5.8 ± 0.3
	Echo 2: 46.1 ± 2.0	Echo 2: 6.3 ± 0.3
	Echo 3: 37.3 ± 1.9	Echo 3: 6.5 ± 0.4

*Note*: Tolerance levels for each sequence are given in Table 2.

Abbreviations: 2D‐MESE, two‐dimensional multi‐echo spin‐echo; MS‐FFE, multi‐slice fast field‐echo; MS‐MESE, multi‐slice multi‐echo spin‐echo.

#### 3.1.2 Spatial linearity

**FIGURE 7:**
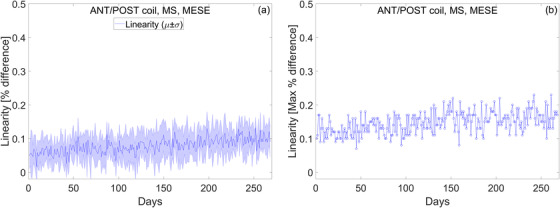
In‐plane geometric distortion is estimated by calculating spatial linearity. (a) The mean and standard deviation of linearity measured across eight radial directions. (b) The maximum deviation of linearity measured across eight radial directions

The longitudinal trend of spatial linearity is shown in Figure 7. Spatial linearity is measured across eight radial directions and is used to estimate in‐plane geometric distortion. Over 1 year, worst‐case linearity is (*μ* ± *σ*) 0.15 ± 0.03% as can be seen in Figure 7b. Spatial linearity was not affected by the noise in the electric components of the couch.

#### 3.1.3 Slice profile

**FIGURE 8:**
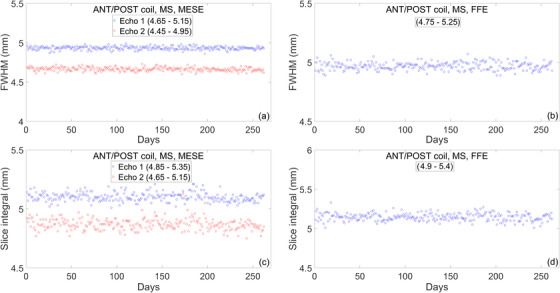
Longitudinal trend of slice profile measurements over the course of 1 year. (a and b) Full width at half maximum (FWHM) measured with the multi‐slice multi‐echo spin‐echo (MS‐MESE) and multi‐slice fast field‐echo (MS‐FFE) sequence. (c and d) Slice integral measured with the MS‐MESE and MS‐FFE sequence

Slice profile measurements over 1 year are presented in Figure 8. The slice profile is characterized by calculating FWHM and slice integral. The mean and standard deviation of the FWHM and slice integral for the first year of clinical use are listed in Table 6. Daily variations for FWHM are on the order of ∼0.6%, as measured by the COV. This worst‐case value is for the second echo of the MS‐MESE sequence. Daily variations for the slice integral are on the order of ∼1%, as measured by the COV. Again, this worst‐case value is for the second echo of the MS‐MESE sequence. FWHM and the slice integral were not affected by the noise in the electric components of the couch.

**TABLE 6:** Mean and standard deviation of the full width at half maximum (FWHM) and slice integral over the course of 1 year

Imaging sequence	FWHM (*μ* ± *σ*) (mm)	Slice integral (*μ* ± *σ*) (mm)
MS‐MESE	Echo 1: 4.93 ± 0.02	Echo 1: 5.10 ± 0.04
	Echo 2: 4.67 ± 0.03	Echo 2: 4.87 ± 0.05
MS‐FFE	4.97 ± 0.03	5.15 ± 0.05

*Note*: Tolerance levels for each sequence are given in Table 3.

Abbreviations: FWHM, full width at half maximum; MS‐FFE, multi‐slice fast field‐echo; MS‐MESE, multi‐slice multi‐echo spin‐echo.

#### 3.1.4 Spatial resolution

**FIGURE 9:**
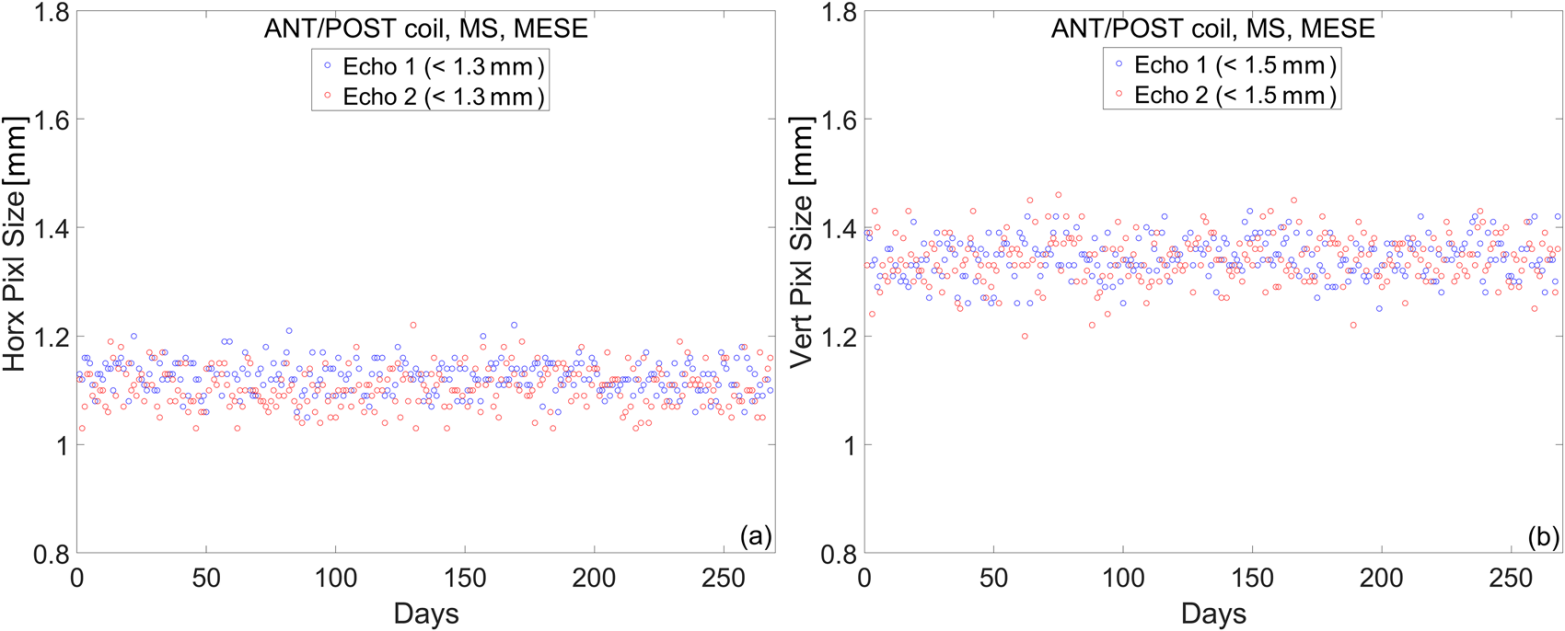
Spatial resolution measurements over the course of 1 year. (a) Horizontal pixel size. (b) Vertical pixel size

Figure 9 shows the longitudinal trend of spatial resolution as measured by the vertical and horizontal pixel size. The mean and standard deviation of these metrics for the first year of clinical use are listed in Table 7. Daily variations for the horizontal pixel size are on the order of ∼4%, as measured by the COV. This worst‐case value is for the second echo of the MS‐MESE sequence. Daily variations for the vertical pixel size are on the order of ∼3%, as measured by the COV. Again, this worst‐case value is for the second echo of the MS‐MESE sequence. Spatial resolution measurements were not affected by the noise in the electric components of the couch.

**TABLE 7:** Mean and standard deviation of the horizontal and vertical pixel size over the course of 1 year

Imaging sequence	Horizontal pixel (mm)	Vertical pixel (mm)
MS‐MESE	Echo 1: 1.12 ± 0.03	Echo 1: 1.34 ± 0.04
	Echo 2: 1.11 ± 0.04	Echo 2: 1.34 ± 0.04

*Note*: Tolerance levels for each echo are given in Table 4.

Abbreviation: MS‐MESE, multi‐slice multi‐echo spin‐echo.

#### 3.1.5 Central frequency

**FIGURE 10:**
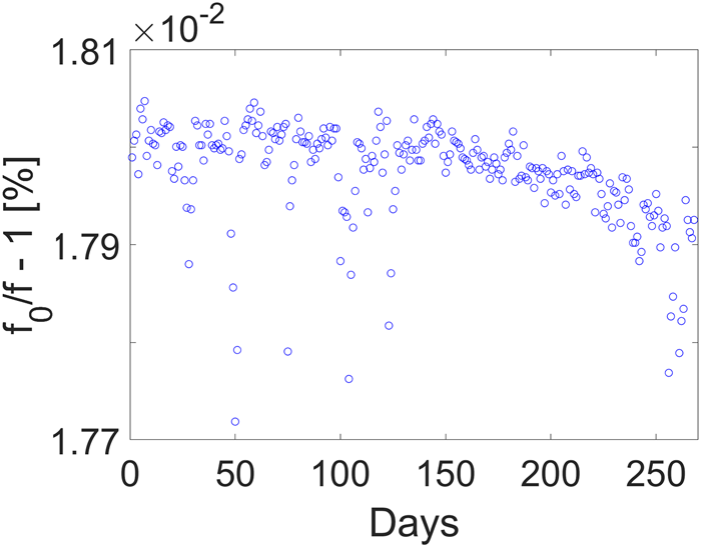
Central frequency measurements over the course of 1 year. Baseline values were approximately 0.018% lower than the nominal with a drift of approximately 0.5% per year

Figure 10 plots the daily variation of the resonant frequency normalized to the nominal value at 1.5 T. At baseline, the central frequency was approximately 0.018% lower than the nominal. Using a linear fit, the B0 drift is estimated to be approximately 0.5% per year. While we present data only for the central frequency measured with the MS‐MESE sequence, we find that the trend is the same when either the MS‐FFE or 2D‐MESE is used for analysis.

### 3.2 Distortion

**FIGURE 11:**
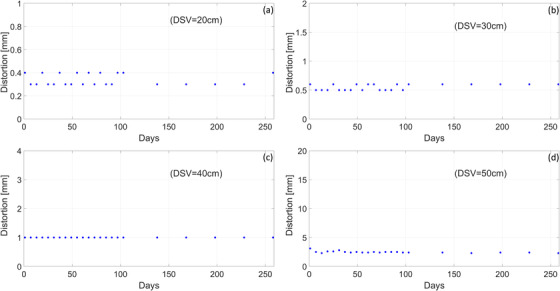
Longitudinal trend of three‐dimensional (3D)‐distortion measurements over the course of 1 year. For each diameter of spherical volume (DSV), the *y*‐axis is shown in the range of the tolerance level. The quality assurance (QA) test was performed weekly during the first three months of clinical use and monthly thereafter. Maximum distortion for (a) DSV = 20 cm, (b) DSV = 30 cm, (c) DSV = 40 cm, and (d) DSV = 50 cm

**FIGURE A3:**
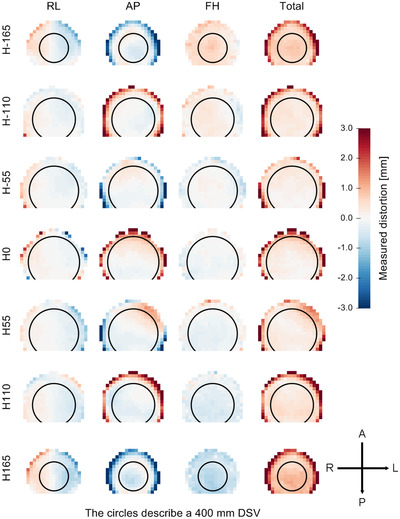
Spatial distribution of geometric distortion measured at the plane of the isocenter and at ±55, ±110, ±165 mm away from the isocenter. The distortion is displayed in the range of ±3 mm. The heatmap is used as a consistency check for qualitative comparisons with baseline measurements. DSV, diameter of spherical volume

The longitudinal trend of 3D‐distortion measurements is shown in Figure 11. The maximum value of distortion is estimated within the region defined by the spherical volume centered at the isocenter. The longitudinal 3D geometric analysis reveals that the maximum distortion in a DSV of 20, 30, 40, and 50 cm is 0.4, 0.6, 1.0, and 3.1 mm, respectively. While the maximum distortion provides a convenient metric for long‐term analysis, the distortion vector field allows for a detailed assessment of its effect and, if needed, for corrections. The magnitude of the distortion field is shown as a heatmap in Figure A3. The heatmap is used as a consistency check for qualitative comparisons with baseline measurements.

### 3.3 B0 and B1 homogeneity

**FIGURE 12:**
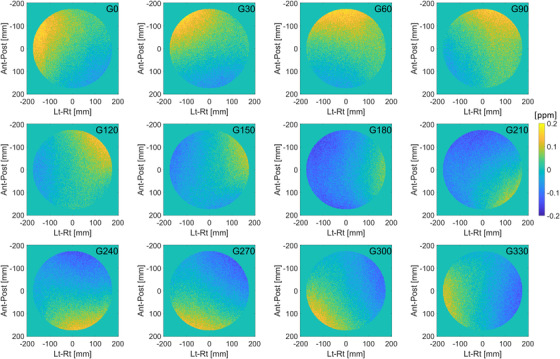
Distribution of B0 as a function of gantry angle. The mean B0‐map across all gantry angles is subtracted from each measurement to remove the static B0 contribution

**FIGURE A4:**
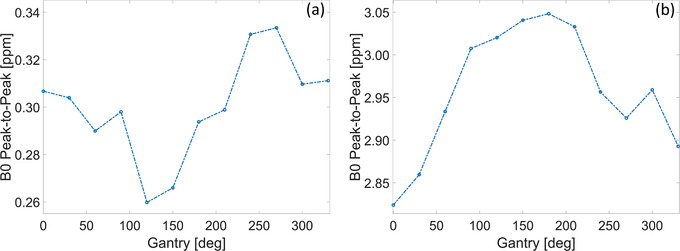
Peak‐to‐peak variation in B0‐maps as a function of gantry angle. (a) The static B0 contribution has been removed to highlight only the gantry dependence. (b) The static B0 contribution has not been removed showing the combined effect with gantry angle. Note that pre‐scan calibration measurements are only acquired at gantry 0° to simulate the worst‐case scenario when magnetic resonance imaging (MRI) imaging begins before and is performed during treatment delivery (e.g., cine acquisition for motion monitoring)

The homogeneity of the B0 and B1 fields can be used both for QA measurements and as a correction factor in quantitative MRI biomarkers. The distribution of B0 as a function of gantry angle is given in Figure 12. The mean B0‐map across all gantry angles is subtracted from each measurement to remove the static B0 contribution. The peak‐to‐peak variation is plotted in Figure A4. When the static B0 contribution is not removed from each measurement, the maximum peak‐to‐peak variation is 3.05 ppm for gantry at 180°. Note that pre‐scan calibration measurements are only acquired at gantry 0° to simulate the worst‐case scenario when MRI imaging is performed during treatment delivery.

**FIGURE 13:**
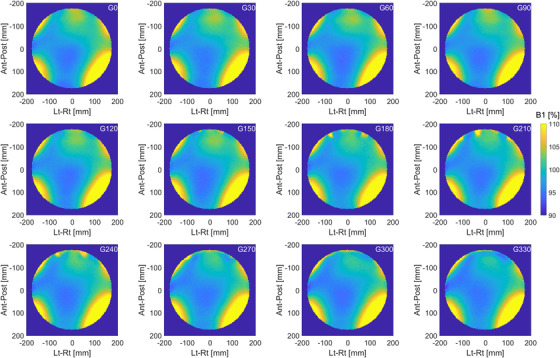
Distribution of B1 as a function of gantry angle. Measurements are performed in a large cylindrical phantom with a diameter of 40 cm and analyzed inside a circular region with a diameter of 35 cm. B1‐maps are shown as a percent of power needed to achieve the prescribed flip angle

**FIGURE A5:**
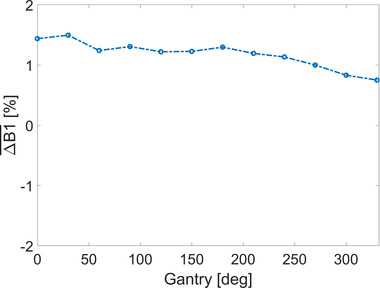
Average percent deviation from the nominal B1 power as a function of gantry angle. Measurements are performed in a large cylindrical phantom with a diameter of 40 cm and analyzed inside a circular region with a diameter of 35 cm

The distribution of B1 as a function of gantry angle is given in Figure 13. B1‐maps are shown as a percent of power needed to achieve the prescribed FA. Figure A5 shows the average percent deviation from the nominal B1 power as a function of gantry angle. Over all angles, the average deviation is 1.2 ± 0.2%, with minimal dependence on gantry position. As in the case of B0 mapping, pre‐scan calibration measurements are only acquired at gantry 0°.

### 3.4 Quantitative MRI

**FIGURE 14:**
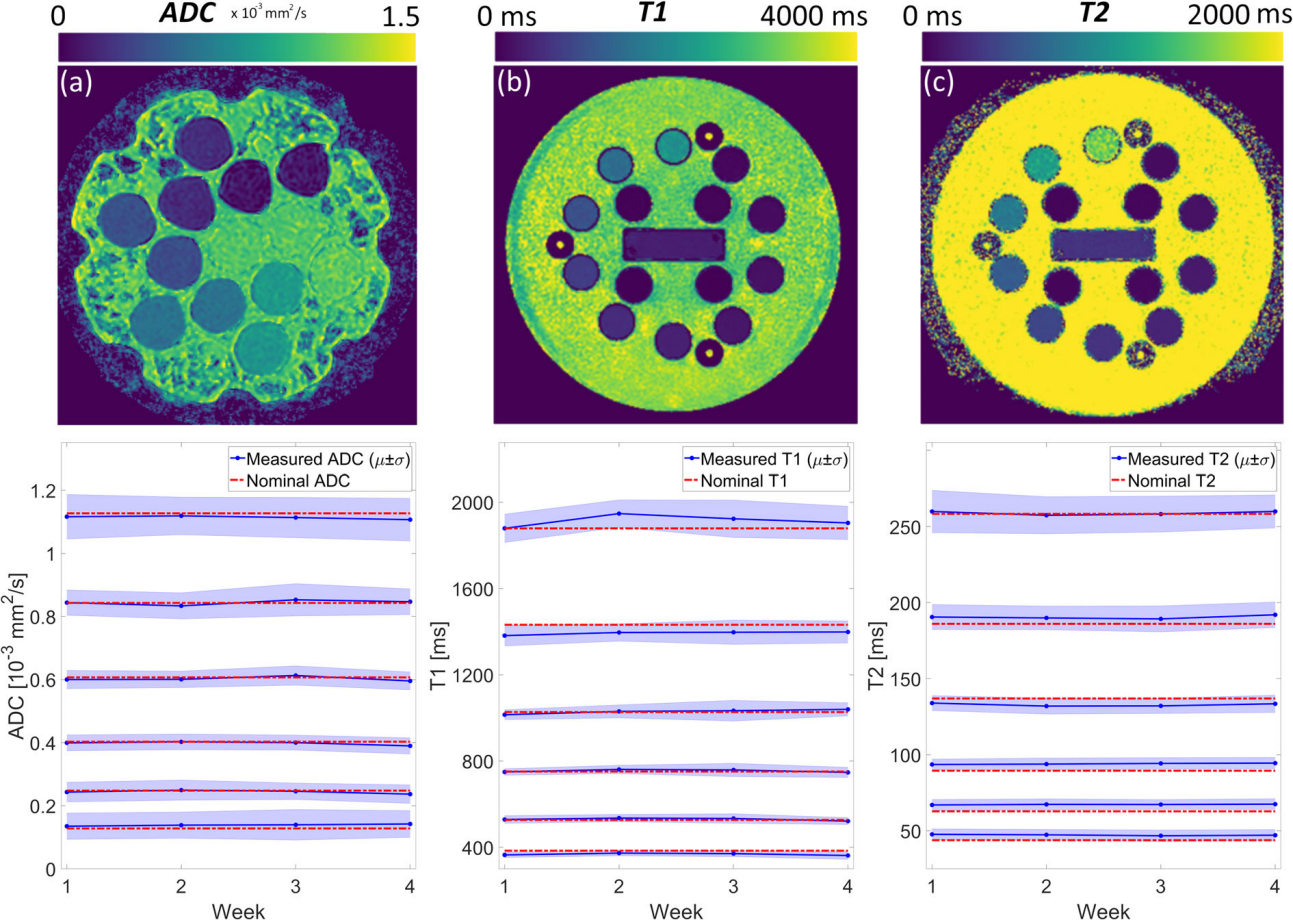
Representative quantitative magnetic resonance imaging (MRI) maps and longitudinal trend for (a) apparent diffusion coefficient (ADC), (b) T1, and (c) T2. Measurements were performed weekly over the course of 4 weeks using two National Institute of Standards and Technology (NIST) traceable phantoms. The mean and standard deviation are measured in a circular region‐of‐interest (ROI) of 1‐cm diameter centered on each vial. Nominal values are given in Tables A1 and A2

Representative maps of ADC, T1, T2, and measurements over the course of 4 weeks are shown in Figure 14. The longitudinal graphs plot the mean and standard deviation in a circular ROI of 1‐cm diameter centered on each vial of interest. Note that for ADC, the values included in the analysis are measured only in the inner ring of vials in the diffusion phantom. The bias and reproducibility for all measurements (for entire range of qMRI biomarkers and all 4 weeks) are given in Table 8. Median bias for ADC, T1, and T2 is −0.8%, −0.1%, 3.9%, and median COV is 1.3%, 1.1%, 0.5%, respectively.

**TABLE 8:** The median (and range) of bias and reproducibility for apparent diffusion coefficient (ADC), T1, and T2 after combining measurements from all 4 weeks

	B (%)	COV (%)
ADC	−0.8 (‐4.5 to 11.1)	1.3 (0.5–2.2)
T1	−0.1 (‐5.8 to 3.6)	1.1 (0.6–1.5)
T2	3.9 (‐3.6 to 8.8)	0.5 (0.3–0.8)

Abbreviation: COV, coefficient of variation.

**FIGURE A6:**
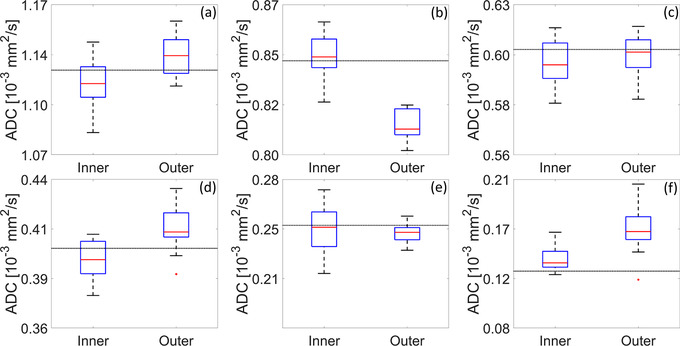
Comparison of apparent diffusion coefficient (ADC) values measured in the inner ring of vials versus the outer ring of vials. Refer to Figure A1 for description of inner/outer ring. For the vials presented in panels (a, b, d, and f), mean ADC values in the inner ring were significantly different from the mean values in the outer ring (*t*‐test, *p* << 0.05). Dashed line depicts the nominal value

Table A3 separates the bias and reproducibility for each of the qMRI biomarkers included in the study. The bias of ADC is not significantly dependent on the value of the measured ADC (Spearman's test, *p* = 0.714), but the COV is strongly dependent on the measured ADC values (Spearman's test, *ρ* = ‐0.94, *p* = 0.017). We find that ADC values are also dependent on distance from isocenter, as seen in Figure A6. In four out of six vials, the mean measured ADC in the inner ring was significantly different from the measured values in the outer ring (*t*‐test, *p* ≪ 0.05). A comparison of the artifacts that arise in ADC maps acquired with the SS‐EPI sequence versus a Cartesian spin‐echo sequence is shown in Figure A1.

For T1, the dependence of bias and COV on the measured T1 values is not significant (Spearman's test, *p* = 0.356 and 0.556, respectively). For T2, the dependence of bias and COV on the measured T2 values is not significant (Spearman's test, *p* = 0.058 and 0.297, respectively).

### 3.5 ACR

Image quality is also evaluated using the large phantom and T1/T2‐weighted sequences with imaging parameters recommended by the ACR. The results are given in Table 9.

**TABLE 9:** Results of image quality assessment using American College of Radiology (ACR) testing recommendations

	T1‐weighted	T2‐weighted	Tolerance
Geometric accuracy	<1 mm	<1 mm	<±2 mm
Slice position accuracy	1.8 mm	1.9 mm	−5 mm < x < 5 mm
Slice thickness	4.8 mm	4.7 mm	5 ± 0.7 mm
PIU	91.4%	91.8%	>87.5%
PSG	0.0012	0.0014	≤0.025
HCSR	0.9	0.9	≤1
LCD	28	22	>9

Abbreviations: HCSR, high‐contrast spatial resolution; LCD, low‐contrast detectability; PIU, percent integral uniformity; PSG, percent signal ghosting.

## 4 DISCUSSION

This report presents the 1‐year longitudinal trend of QA measurements for one of the earliest Elekta Unity MR‐linacs in clinical use in the United States. The focus of this work is on performance and stability of the MRI component of the machine. The analysis of the QA measurements for the linac component can be found in the literature.^21^

Currently, routine MRI QA is primarily concerned with the quality of images acquired with protocols recommended by vendors and accreditation organizations. Image quality is quantified by measuring or calculating specific metrics and comparing to published tolerance levels. For the Unity system, the tolerances for image QA are provided by the MRI vendor and emphasized in a recent report by the Elekta MR‐linac working group.^22^ Standardized acquisition methods, analyses, and tolerance levels are still needed for quantitative MRI biomarkers measured in the MR‐linac.

The PIQT methods have been designed for diagnostic scanners and MR‐simulators. As such, there is either a one‐to‐one correspondence with ACR tests (e.g., SNR, slice thickness, uniformity) or a correlation between metrics can be deduced (e.g., pixel size, resolution, detectability). PIQT allows for a convenient phantom setup for daily QA, automatic image analysis, and accessible long‐term storage of the raw data. However, the phantom is intended for use only in Philips MR scanners and cannot be readily translated across vendors. The 1‐year assessment of PIQT measurements reveals that the Unity system operates within the guidelines and recommendations for scanner performance and stability.^22^ Our analysis serves as an independent evaluation of the suggested tolerance levels. Daily variations in each quality metric are well below the threshold for failure. While the SNR is an aggregate and non‐specific measure of system performance, it remains a key parameter in evaluating the entire imaging chain. The electronic noise originating in the treatment couch was only detected by the gradual drop in SNR, reinforcing the need for daily MR QA. All other parameters were not affected. Note that the vendor specifies a frequency of daily measurements for SNR and weekly for PIQT. Early users may find it useful to initially run PIQT daily to generate baseline data for their scanners.

Geometric distortion is of particular concern in radiotherapy. Linearity measurements in PIQT estimate in‐plane distortion but provide limited information on its spatial distribution. The longitudinal 3D geometric analysis in Figure 11 reveals that the maximum distortion in a DSV of 20, 30, 40, and 50 cm is 0.4, 0.6, 1.0, and 3.1 mm, respectively. As expected, the magnitude of total distortion is negligible near the isocenter and increases roughly symmetrically as a function of distance from the isocenter, as seen in Figure A3. Nevertheless, distortion is not symmetric along the cardinal axes and an analysis of the spatial distribution is needed for longitudinal consistency checks. The Elekta MR‐linac working group recommends only the 3D geometric distortion test for monthly MRI QA.^22^

There are currently no consensus recommendations for annual imaging QA in Unity. We have decided to perform annual ACR testing even though the scanner is not intended for diagnostic use. Table 9 shows that MRI performance in the Unity MR‐linac is comparable to diagnostic scanners despite radical differences in system design.^19^ Other institutions have reported similar findings.^18^, ^19^, ^38^

The bias and uncertainty for qMRI biomarkers depends on system parameters, acquisition methods, and biomarker estimation model. In addition to PIQT and ACR metrics, main and excitation field uniformity are essential system parameters affecting the accuracy of every qMRI measurement. In our system, the maximum peak‐to‐peak B0 inhomogeneity is found to be ∼3 ppm. Given a field inhomogeneity ΔB0 and read gradient strength of *G*_read_, the magnitude of distortion can be calculated by ΔB0/*G*_read_. Using the maximum gradient strength in Unity of 15 mT/m, the maximum B0‐distortion is ∼0.3 mm. In context, the smallest voxel size in the approved clinical exam cards is 1.0 × 1.0 × 1.0 mm^3^. Furthermore, operation under the maximum gradient strength is avoided for safety reasons. Gantry induced B0‐distortions are an order of magnitude smaller, as seen in Figure A4. A comparison with the magnitude of total distortion in Figure 11 shows that the main contribution to geometric distortion is from gradient nonlinearity. Therefore, the total distortion measurement presented in this paper is an estimate of total distortion for other sequences using 3D spatial encoding. This has been confirmed with a 3D T2‐weighted spin‐echo acquisition that we use for online plan adaptation.

The average deviation from the nominal B1 is within 2% in an ROI of 35 cm diameter, with minimal dependence on gantry angle. The distribution of the B1 field is dependent on the shape and electromagnetic properties of the object being scanned, resonance frequency (field strength), RF‐pulse design (hard vs. slice selective or adiabatic), and the polarization of the excitation coil.^39^ The large uniform phantom provides a convenient setup for simultaneous checks of long‐term B0 and B1 uniformity.

Mean ADC, T1, and T2 values are measured with high reproducibility in the Elekta Unity MR‐linac. When combining the measurements from all 4 weeks, the COV for ADC, T1, and T2 is 1.3%, 1.1%, and 0.5%, respectively. The bias for ADC, T1, and T2 is −0.8%, −0.1%, and 3.9%. Kooreman et al.^27^ report a consistently larger bias for T2 values as well. Note that for single‐institutional studies, reproducibility takes priority over bias, especially for relaxometry biomarkers for which the biological interpretation remains unclear. In our system, worst‐case longitudinal repeatability for ADC, T1, and T2 is 2.5%, 2.9%, and 1.5%, respectively, as shown inTable A3. Therefore, we consider a 3% mean difference with respect to baseline as the threshold for tolerance of qMRI QA testing. In the context of multi‐institutional studies, the bias would be of equal importance to repeatability. Initially, weekly qMRI QA measurements may be necessary to establish the baseline for bias and repeatability cross‐comparisons. This may be followed by monthly QA measurements, then only annually and as needed after machine servicing.

Improved methods for quantitative MRI will decrease the bias and variability of the estimated biomarkers. In Figure A1 we provide an example of ADC mapping with a Cartesian spin‐echo sequence where the effect of susceptibility distortions is minimized. Long‐term bias and uncertainty measurements with the Cartesian spin‐echo sequence need further investigation.^40^ Improved methods for relaxometry have also been recently demonstrated in the Unity system.^41^

## 5 CONCLUSION

We report the 1‐year longitudinal trend of MRI QA measurements for an Elekta Unity machine in clinical use in our institution. Our findings show that the MRI component operates within the guidelines and recommendations for scanner performance and stability.^22^ The analysis of the data supports the recently published guidance in establishing clinically acceptable tolerance levels for image quality.

## AUTHOR CONTRIBUTIONS

All authors contributed to the conception and design of the work, drafting, and final approval of manuscript.

## CONFLICT OF INTEREST

The authors declare they no conflict of interest.

## APPENDIX A

### A.1

**TABLE A1:** Nominal T1 and T2 values in the National Institute of Standards and Technology (NIST)/International Society of Magnetic Resonance in Medicine (ISMRM) phantom used in this study

T1 (ms)	T2 (ms)
1879.0	1044.0
1432.0	623.9
1027.0	428.3
751.3	258.4
527.0	186.1
384.1	137.0
272.3	89.52
194.5	62.82
137.8	43.84
94.7	27.28
67.0	19.24
48.14	15.44
34.35	10.05
24.16	7.79

**TABLE A2:** Concentration of polyvinylpyrrolidone (PVP) and nominal apparent diffusion coefficient (ADC) values at 0°C

PVP concentration (%)	Nominal ADC (10^−3^ mm^2^/s)
0	1.127
10	0.843
20	0.607
30	0.403
40	0.248
50	0.128

**TABLE A3:** Bias and reproducibility of longitudinal measurements over 4 weeks

ADC_n_ (10^−3^ mm^2^/s)	$\mu_{L}$	${\tilde{\mu }}_{L}$	$\sigma_{L}$	$R_{L}$	$B_{L}$ (%)	COV_L_ (%)
1.127	1.114	1.115	0.006	0.012	−1.2	0.5
0.843	0.845	0.846	0.009	0.020	0.2	1.1
0.607	0.603	0.601	0.008	0.018	−0.7	1.3
0.403	0.399	0.400	0.006	0.013	−1.0	1.5
0.248	0.244	0.245	0.006	0.013	−1.6	2.5
0.128	0.139	0.140	0.003	0.008	8.6	2.2

T1_n_ (ms)						
1879.0	1895.9	1906.4	44.7	150.4	0.9	2.4
1432.0	1385.5	1396.7	28.9	93.6	−3.2	2.1
1027.0	1022.4	1025.1	17.2	45.2	−0.4	1.7
751.3	750.5	752.6	15.7	47.2	−0.1	2.1
527.0	525.7	527.7	11.4	32.9	−0.2	2.2
384.1	362.8	363.4	10.7	31.5	−5.5	2.9

T2_n_ (ms)						
258.4	259.4	259.9	4.0	12.6	0.4	1.5
186.1	190.2	190.3	1.7	5.2	2.2	0.9
137.0	133.1	133.0	1.7	5.8	−2.8	1.3
89.5	94.2	94.4	0.8	2.3	5.3	0.8
62.8	67.5	67.5	0.5	1.3	7.5	0.7
43.8	47.4	47.6	0.5	1.2	8.2	1.1

*Note*: ADC_n_/T1_n_/T2_n_, nominal apparent diffusion coefficient (ADC)/T1/T2; $\mu_{L}$/${\tilde{\mu }}_{L}$/$\sigma_{L}$/$R_{L}$, mean/median/standard deviation/range of longitudinal measurements (in units of their respective quantitative magnetic resonance imaging (qMRI) biomarker); $B_{L}$/COV_L_, bias and coefficient of variation of longitudinal measurements.
